# Recurrent bullous eruptions predominantly involving previously treated breast cancer sites

**DOI:** 10.1016/j.jdcr.2026.06.029

**Published:** 2026-06-19

**Authors:** Mizuho Namba, Kazuhito Kogawa, Akari Terada, Yasushi Matsuzaki, Daiki Rokunohe, Takaya Murai, Eijiro Akasaka

**Affiliations:** a Department of Dermatology, Hirosaki University Graduate School of Medicine, Hirosaki, Japan; b Department of Dermatology, Hachinohe City Hospital, Hachinohe, Japan

**Keywords:** BP180, BP230, bullous pemphigoid, radiation therapy

## Case description

**Fig 1:**
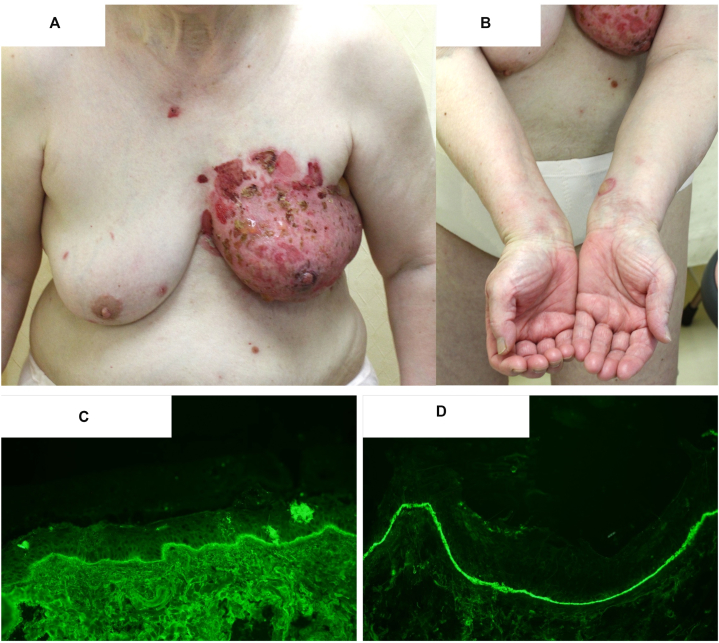
**A** and **B,** Multiple skin erosions and blisters confined to the left breast. A few blisters appeared on the forearm and back. **C** and **D,** Direct immunofluorescence. Deposition of immunoglobulin G (IgG) **(C)** and C3 **(D)** along the basement membrane (direct immunofluorescence, original magnification ×40).

A 74-year-old female patient underwent surgery for invasive ductal carcinoma of the left breast, followed by adjuvant radiotherapy (50 Gy in 25 fractions) and hormone therapy with letrozole (Month 0). One month after completion of radiotherapy, a few blisters developed at the irradiated site on the left breast and gradually increased. At Month 4, immunoglobulin G autoantibodies to the NC16A domain of BP180 (anti-BP180NC16A) in chemiluminescent enzyme immunoassay were negative. Topical corticosteroids were initiated under the diagnosis of radiation dermatitis, and the eruption resolved completely. At Month 11, new blisters appeared on the left breast, and then they had expanded to the limb and back in 1 month (Fig 1, *A* and *B*). Anti-BP180NC16A antibody levels slightly increased to 12.4 U/mL (normal values <9.0 U/mL) in chemiluminescent enzyme immunoassay at the time. A skin biopsy obtained from erythema on the forearm revealed vacuolar degeneration at the epidermal basal lamina. Direct immunofluorescence demonstrated linear deposition of immunoglobulin G and C3 along the basement membrane (Fig 1, *C* and *D*). At Month 13, oral prednisolone was started at 30 mg/day (0.4 mg/kg/day), leading to complete resolution of eruptions within 1 month. The prednisolone dose has been gradually tapered. At Month 34, the anti-BP230 antibody was measured for the first time using enzyme-linked immunosorbent assay, revealing an elevated level of 55.5 U/mL (normal values <9.0) even though no blister was present. The patient showed a good response to oral prednisolone throughout the clinical course. Although occasional relapses were observed (Month 44), the overall disease course was relatively mild. The anti-BP180NC16A antibody in chemiluminescent enzyme immunoassay had been negative after the first positive result and has remained undetectable through the present (Month 66).

**Question: Which of the following is the most likely diagnosis?**

**A.** Radiation dermatitis
**B.** Drug eruption of letrozole
**C.** Relapse of breast cancer
**D.** Radiation-induced bullous pemphigoid (BP)
**E.** Paraneoplastic pemphigus

## Discussion

Correct answer: D. Radiation-induced BP.

BP is the most frequent autoimmune bullous disease, caused by autoantibodies targeting hemidesmosomal plaque protein BP180 and/or BP230. The majority of BP cases present as generalized cutaneous disease. However, only a limited number of BP patients develop localized bullous eruptions. Localized BP can be occasionally triggered by radiation therapy.

Nguyen reported that 93% of radiation-induced BP cases initially developed at the site of irradiation, while 41% subsequently extended to other sites.^1^ In our case, generalized eruptions coincided with a mild increase in anti-BP180 antibodies (Month 12). Notably, anti-BP180 antibodies were undetected during the initial blister development (Month 4) and the first local recurrence (Month 29). This suggests that anti-BP180 antibodies may play a role in the transition from localized BP to systemic BP. On the other hand, anti-BP230 antibody levels remained elevated after clinical remission (Month 34), which was followed by disease relapse (Month 44). Hayakawa reported that anti-BP230 antibodies alone induce mild and localized BP.^2^ Furthermore, Thoma-Uszynski reported BP230 is associated with localized forms of BP.^3^ These findings may suggest a potential relationship between persistently elevated anti-BP230 antibody levels and an increased risk of localized recurrence. Although a recent report does not generally support the use of anti-BP230 antibodies for routine disease monitoring,^4^ longitudinal assessment of anti-BP230 antibody levels may be clinically relevant, particularly in patients with localized BP.

## Conflicts of interest

None disclosed.
